# Enhancing student confidence and preparedness in pharmacology: a virtual pre-laboratory approach

**DOI:** 10.3389/fphar.2026.1897899

**Published:** 2026-10-26

**Authors:** Alexander C. Voisey, Owen J. Crawford, Andrew Hilbourne, Nicola J. Jordan, Iain R. Hutcheson, Derek Lang

**Affiliations:** 1 Medical Pharmacology, School of Pharmacy and Pharmaceutical Sciences, Cardiff University, Cardiff, United Kingdom; 2 Cardiff Learning and Teaching Academy, Cardiff University, Cardiff, United Kingdom

**Keywords:** pharmacology education, pre-laboratory learning, simulation-based learning, student confidence, student experience, student preparedness, technology-enhanced learning, virtual laboratory

## Abstract

**Introduction:**

Laboratory practicals are a core component of undergraduate pharmacology education but are often associated with student anxiety due to lack of familiarity with the lab environment and experimental procedures. Virtual pre-laboratory activities have the potential to address this challenge by allowing students to familiarise themselves with the practical setting and procedures in advance of in-person classes.

**Methods:**

A virtual, interactive desktop-based pre-laboratory resource (VR-Lab) was developed to support second year BSc Medical Pharmacology students undertaking *in-vitro* organ bath experiments. The VR-lab incorporated a 360-degree laboratory environment, stepwise procedural guidance and formative activities. A mixed-methods evaluation was conducted with a cohort of 34 students using Likert-scale survey responses and open-ended feedback following the first practical session. Comparisons of summative assessment performance were made with a previous cohort without access to the VR-Lab.

**Results:**

Students reported high levels of engagement, usability and perceived educational value. All respondents reported the VR-lab enhanced preparedness for and confidence during the in-person laboratory session. Qualitative feedback highlighted the importance of procedural familiarisation, stepwise guidance, and embedded multimedia content in supporting engagement. Feedback also identified areas for improvement included navigation and accessibility on smaller devices. No significant difference in summative assessment performance was observed between cohorts.

**Conclusion:**

Virtual pre-laboratory resources may enhance the student experience of practical pharmacology teaching by reducing barriers associated with unfamiliar environments, supporting confidence and engagement without directly influencing assessment outcomes.

## 1 Introduction

Hands-on laboratory practical classes are an essential component of undergraduate pharmacology education (Salmon, 2013), ensuring students develop core skills aligned with sector values (British Pharmacological Society, 2021). Importantly they take numerous forms, covering a wide range of applications ranging from bioassays, effects of drugs on isolated tissues to *in-vivo* models (Curry and Whelpton, 1983; Salmon, 2013; Carriere et al., 2023; Wilhelmus and Drukarch, 2025). These practicals are designed to familiarise students with good laboratory practice, imitating the way professional scientists carry out an experiment (Kirschner, 1992). Although this ‘learning by doing’ approach effectively contributes to student learning processes (Kolb, 2014), for many these practical classes cause significant anxiety (Kurbanoglu and Akin, 2010; Coyte, 2023). Lack of familiarity with equipment, time-pressure and links to assessment are among the challenges that can hinder student confidence and preparedness, reducing student engagement with the experience (Ural, 2016; Coyte, 2023; Wilhelmus and Drukarch, 2025).

Digital technologies, including simulations have been used for many years in pharmacology and related disciplines to address these issues (Rayamajhi et al., 2024). While such approaches can improve familiarity with underlying concepts, they often lack alignment with the specific experimental setup and workflow that students encounter in the laboratory. For example, organ bath investigation of isolated tissues, is central to the development of undergraduate pharmacologist education (Salmon, 2013). While there are numerous existing and freely downloadable digital simulations (such as OBSim (Dempster, 2020)) that aim to replicate organ bath experimentation they prioritise conceptual understanding at the expense of procedural context. This limits their ability to reduce uncertainty around equipment handling, experimental sequencing, and practical logistics.

Virtual pre-laboratory activities have the potential to bridge this gap, enhancing student preparedness by allowing learners to explore experimental environments and procedures in advance of scheduled laboratory sessions (George-Williams et al., 2022). When designed to closely reflect authentic laboratory practice, “virtual pre-labs” may reduce cognitive load during subsequent practical sessions (Girmay et al., 2026), enabling students to focus more effectively on data interpretation and experimental reasoning. While the value of such interventions may not be a direct impact on assessment performance, improvements to student confidence, engagement, and perceived readiness to participate in laboratory activities are key outcomes (Carroll et al., 2025; Griffin et al., 2025).

In the current study we describe the development and evaluation of an interactive desktop-based 360-degree virtual pre-lab simulation (VR-Lab) experience designed to support second year BSc Medical Pharmacology students undertaking *in-vitro* organ bath experiments. In contrast to existing digital simulations that focus on conceptual understanding, the VR-Lab was designed to mirror the physical laboratory environment and utilise a stepwise approach to familiarise students with the experimental protocol in advance of practical classes. Here we evaluate student perceptions of preparedness, confidence and usability to provide evidence for the use of low-cost, scalable virtual pre-lab activities as a supportive tool in pharmacology education.

## 2 Methods

### 2.1 Resource design and development

A 360-degree virtual and interactive desktop-based pre-laboratory resource (VR-Lab) was developed to enhance learning within a second-year undergraduate Medical Pharmacology module ‘Application of *In-Vitro* Pharmacology in Research’ at Cardiff University. The resource was designed to complement existing laboratory teaching by familiarising students with the experimental environment, equipment, and workflow prior to attending the practical session. Development focused on supporting student preparedness and confidence rather than replacing hands-on laboratory skill acquisition.

The resource combined 360-degree photographs in 3dVista with the e-learning package XERTE, both of which are no-code authoring platforms. The use of 360-degree images allowed the physical layout of the laboratory and key pieces of equipment to be represented authentically (Figure 1A). Interactive components were embedded via XERTE to guide students through the experimental sequence using a stepwise approach, incorporating explanatory text (Figure 1B) and short instructional videos at key stages of the workflow (Figure 1C). A formative quiz element was integrated at a relevant stage to support drug concentration calculations required to carry out the experiment (Figure 1D). This format enabled content to be refined iteratively during development.

**FIGURE 1:**
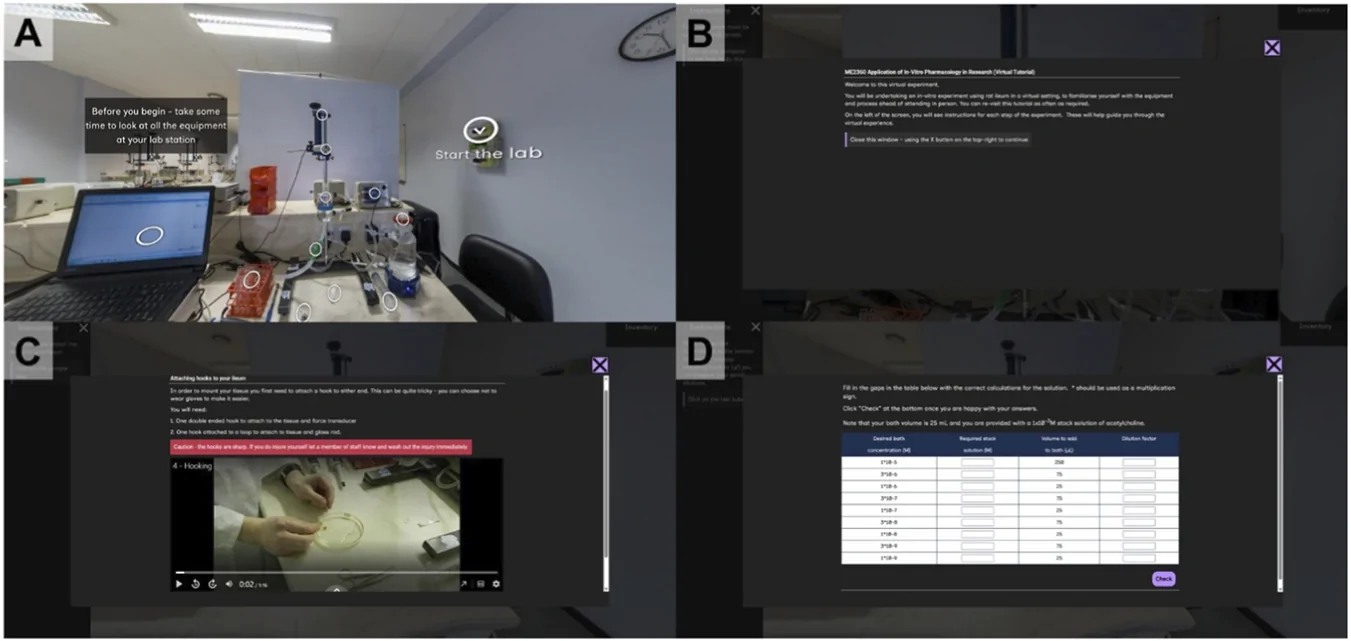
Representative screenshots of the VR-Lab highlighting key features. **(A)** Physical lab layout and equipment used. **(B)** Example of explanatory text. **(C)** Example of embedded instructional videos. **(D)** Example of formative quiz element.

To create the VR-Lab, a series of 360-degree photographs representing each stage of the experimental protocol were captured and arranged within 3dVista to recreate the practical workflow (Supplementary Figures S1 and S2). Interactive hotspots were positioned over relevant equipment at each stage in the protocol allowing students to progress sequentially through key experimental steps in the correct order. For example, students selected the appropriate pipette, transferred drug solutions to a test tube, and subsequently added the drug to the organ bath, mirroring the workflow undertaken during the in-person practical. Instructional overlays and an interactive inventory system were created using XERTE and integrated within the 360-degree environment. These overlays incorporated demonstration videos created using Panopto, providing procedural guidance throughout the activity.

The VR-Lab was designed as a browser-based desktop resource and did not require immersive virtual reality hardware, allowing students to access the resource using a standard web browser on desktop and mobile devices.

### 2.2 Resource delivery

The VR-Lab was made available to all students (n = 34) enrolled on the module in the 2024/25 academic year via Blackboard Ultra. Students were provided with a link to the VR-Lab prior to their first practical class and access remained available for the duration of the module. Students were encouraged to engage with the resource in advance of the first laboratory session; however, completion was not compulsory and was not linked to assessment. The VR-Lab was explicitly framed as a preparatory tool to enhance familiarity with the experimental apparatus and procedure. Student engagement with the VR-lab was monitored using the Student Progress function of Blackboard Ultra across the duration of the module.

### 2.3 Resource evaluation

Evaluation of the virtual pre-laboratory resource was conducted anonymously using Microsoft Forms. The voluntary survey was made available to students immediately following completion of the first practical class of the module. The survey comprised of three sections. Student perceptions were initially gathered using five-point Likert-scale questions (1 = Strongly Disagree – 5 = Strongly Agree). Questions assessed engagement, user experience, preparedness, confidence and perceived educational value (Supplementary Material). A singular binary question was also utilised to determine where students encountered technical difficulties with the resource. Finally, three open-ended questions were used to gather qualitative feedback about the student experience. Open-ended responses were analysed qualitatively to identify recurring themes relating to any perceived benefits, user-friendliness and areas for refinement. Likert-scale questions were analysed quantitatively to summarise student perceptions of the resource.

### 2.4 Ethics and consent

Formal research ethical approval was not required as this was classified as evaluation of teaching practice in accordance with guidance provided by the School of Medicine Research Committee at Cardiff University. Participation in the survey was entirely voluntary and anonymous, therefore no identifiable data was collected. Completion of the survey was considered to indicate consent for the inclusion of anonymised responses in the analysis. No direct quotes from survey participants are included in the manuscript. Anonymised assessment data used for cohort-level comparisons were obtained retrospectively and analysed in aggregate form. No individual student or assessment could be identified from the data.

### 2.5 Module structure and assessment

‘Application of *In-Vitro* Pharmacology in Research’ is a 20-credit module that spans 8 weeks across the Spring semester. The module is compulsory for all second year BSc Medical Pharmacology Students at Cardiff University and is comprised of six in-person tutorials and four in-person practical classes (Table 1). Across the four practical classes students will complete four non-cumulative concentration response curves to the muscarinic acetylcholine receptor agonists acetylcholine and carbachol alone and the in the presence of three “mystery drugs”, whose identity is known to staff but not students. In the final practical class students perform a Schild analysis experiment to calculate the pA_2_ value of atropine.

**TABLE 1:** Tutorials and practical classes in ‘Application of *In-Vitro* Pharmacology in Research’.

Tutorial		Practical class	
1	Introduction, equipment and protocol familiarisation, measuring responses	1	Acetylcholine and carbachol non-cumulative concentration response curve, effect of mystery drug 1
2	Isometric tension, roles of Ca^2+^ in smooth muscle contraction, conditioning	2	Acetylcholine and carbachol non-cumulative concentration response curve, effect of mystery drug 2
3	Review practical 1, data interpretation, spontaneous activity, statistical analysis	3	Acetylcholine and carbachol non-cumulative concentration response curve, effect of mystery drug 3
4	Review practical 2, use of animals in research and the 3Rs	4	Acetylcholine and carbachol non-cumulative concentration response curve, pA_2_ of atropine
5	Data interpretation and using Graphpad Prism	​	
6	Review practical 3, Schild analysis and pA_2_ values		

Students are assessed by a dissertation written in the style of a British Journal of Pharmacology scientific paper. They are expected to use analytical skills, critical insight and the wider scientific literature to determine the identities of the mystery drugs.

### 2.6 Data analysis

Engagement with the VR-Lab was determined using access data generated via the Blackboard Ultra Student Progress function. These data were used to summarise student uptake of the resource prior to the practical session.

Quantitative survey responses were collected using five-point Likert-scale, questions were analysed in Microsoft Excel. Response frequencies and percentages were calculated for each item to summarise student perceptions of engagement, usability, preparedness, confidence, and perceived educational value of the VR-Lab.

Qualitative data from open-ended survey questions were analysed using thematic analysis in Microsoft Excel. Responses were reviewed in full, and recurring ideas were initially coded. Codes were refined and grouped into themes representing perceived benefits, usability considerations, and areas for refinement. The frequency of each theme was then calculated.

To explore potential impacts of the VR-Lab on academic performance, anonymised assessment results from the 2024–2025 cohort (with access to the VR-Lab) were compared with those from the 2023–2024 cohort (without access). Mean assessment scores were compared between cohorts using an unpaired two-tailed Student’s t-test.

## 3 Results

### 3.1 Participant engagement

The VR-Lab was made available to the entire Year 2 Medical Pharmacology cohort (34 students) prior to the first organ bath practical. 31 of 34 students completed the VR-Lab prior to attending the first practical class. The remaining three students did not access the resource at any point during the duration of the module. Feedback was obtained from the 10 students who completed the post-practical VR-Lab evaluation survey.

### 3.2 Student perceptions of the VR-Lab

Likert scales were used to determine student perceptions of the VR-Lab (Figure 2) Obtained responses indicate high levels of agreement across evaluated domains. Respondents found the VR-Lab was engaging (mean score 5, strongly agree), easy to navigate (mean score 4.6, strongly agree) and reinforced prior learning (mean score 5, strongly agree). All respondents strongly agreed that the resource was a useful addition to the module and a good example of technology-enhanced learning. While perceived preparedness was uniformly high (mean score 5, strongly agree), responses relating to confidence during the practical session showed a greater degree of variation with 30% of students indicating agreement rather than strong agreement (70%).

**FIGURE 2:**
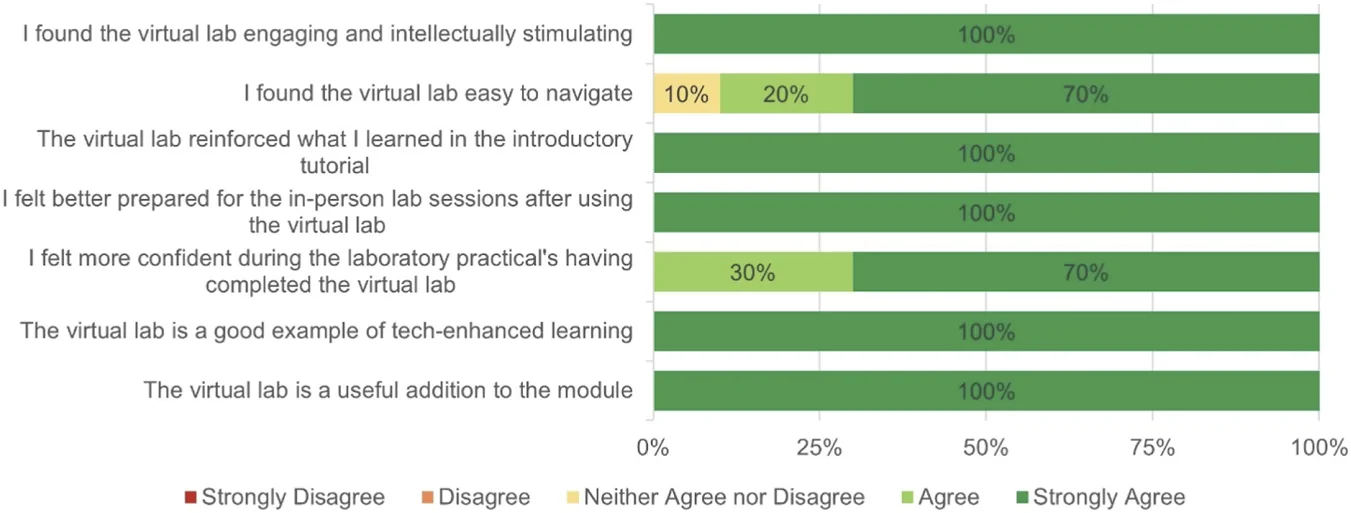
Likert scale rating of student perceptions from survey respondents (n = 10).

### 3.3 Qualitative themes from student feedback

Analysis of open-ended free-text survey responses identified four qualitative themes reflecting the perceived benefits of the resource and areas for improvement (Figure 3). The most commonly identified theme related to enhanced preparedness, familiarity and confidence before and during the practical class. Students stated the VR-Lab was useful for understanding the experimental set up and protocol before entering the physical laboratory, making them feel more confident before and during the class. Additionally, students found the VR-Lab helped them make the most of their time during the in-person class as they spent less time interpreting the experimental protocol or waiting for assistance from academic staff.

**FIGURE 3:**
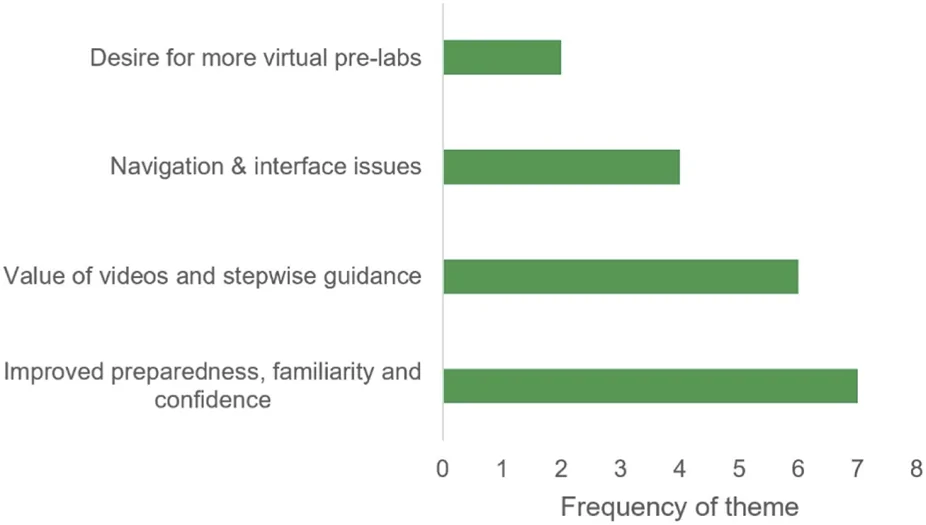
Frequency of identified themes from open-ended student feedback.

The value of videos and stepwise guidance was frequently reported, with students highlighting the usefulness of explanatory content to understand the rationale behind each step in the experimental procedure.

Navigation and interface issues were a common negative perception of the resource. These were primarily linked to screen layout or use on smaller devices such as tablets or smartphones. Suggestions to improve navigational issues included more obvious visual cues and the ability to revisit previous steps.

Finally, a small subset of respondents expressed a desire for similar VR-Labs to be implemented across other in-person practical sessions within the course.

### 3.4 Impact on academic performance

The introduction of the VR-Lab had no impact on the academic performance of students in the summative assessment linked to the module compared to those from the previous academic year (Figure 4). The mean performance in the 2024/25 academic year was 60.2% and not significantly lower than that of the 2023/24 academic year (62.3%). In the 2024/25 academic year there was a single non-submission of the assessment, exclusion of that results in a mean performance 62.2%, again not different to the 2023/24 academic year.

**FIGURE 4:**
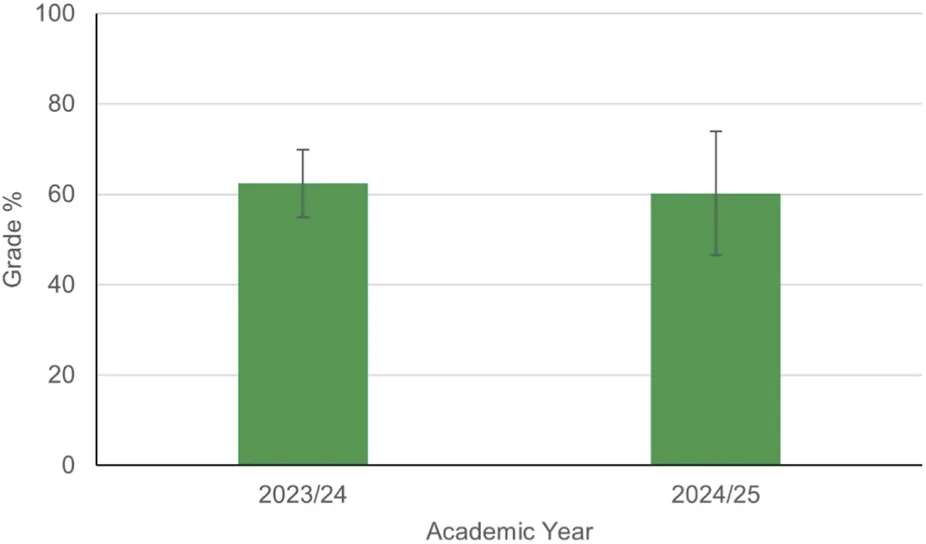
Academic performance of students without (2023/24, n = 44) and with (2024/25, n = 34) access to the VR-Lab.

## 4 Discussion

The current study describes the development and evaluation of a virtual pre-lab resource (VR-Lab) designed to support student preparedness and confidence during *in-vitro* organ bath experimentation in undergraduate pharmacology education. Analysis of student feedback demonstrates the resource was well received by students and perceived as a valuable preparatory tool. Qualitative and quantitative feedback showed that students found the resource was engaging and generally easy to use. There was strong agreement among respondents that the VR-Lab enhanced preparedness for the practical session and increased confidence in completing experimental activities. In contrast the VR-Lab had no impact on the cohort’s summative assessment performance indicating the impact relates purely to student experience rather than academic achievement.

### 4.1 Confidence and preparedness as educational outcomes

Practical laboratory classes play a central role in undergraduate pharmacology education, providing students with opportunities to develop experimental skills required for pharmacological careers (Salmon, 2013; British Pharmacological Society, 2021). However, such activities are also widely recognised as sources of student anxiety, particularly when students are unfamiliar with laboratory environments and equipment, face time-pressured workflows, or perceive close links between practical performance and assessment outcomes (Kurbanoglu and Akin, 2010; Ural, 2016; Coyte, 2023; Wilhelmus and Drukarch, 2025). Such factors can undermine student confidence prior to laboratory sessions, shaping their perceptions of practical classes and limiting their engagement with authentic experiential learning activities.

Given the diverse learner base, the impact of unfamiliar learning environments is unlikely to be equal across a student cohort. Practical class environments often introduce a high sensory demand, that could act as a disproportionate barrier to some learners, further impacting their ability to engage. Simulation and video supported learning environments can help support inclusive practice by allowing learners to rehearse activities in advance and reduce uncertainty around the live setting (Frei-Landau et al., 2026).

The VR-Lab provides a safe and supportive environment in which students can familiarise themselves with the laboratory space, equipment, experimental sequences, and expectations in advance of hands-on work. Student feedback indicated that this advance familiarisation helped them make more effective use of in-person practical time, reduced reliance on academic staff for procedural guidance, and supported greater confidence during laboratory sessions. As such, the VR-Lab addresses environmental and procedural barriers to participation, enabling students to approach practical classes with increased confidence and focus (George-Williams et al., 2022; Girmay et al., 2026).

### 4.2 Academic performance and assessment outcomes

The absence of a measurable difference in summative assessment outcomes between cohorts is consistent with the design and intended role of the VR-Lab. The resource was not designed to align directly with assessment of the module but to support students experience of hands-on laboratory teaching through improved procedural preparedness and confidence. In contrast, the summative assessment required students to demonstrate higher-order skills, including data interpretation, critical evaluation and scientific writing. Therefore, whilst the VR-Lab may have enhanced the practical learning experience, assessment outcomes would not be expected to be sensitive to the type of preparatory intervention evaluated in this study.

### 4.3 Limitations and future directions

This study represents an evaluation of teaching practice within a single undergraduate pharmacology module, and findings should be interpreted within this context. The number of survey respondents was limited, and participation in the evaluation survey was voluntary, introducing potential response bias. Students with a positive experience of the VR-Lab may have been more likely to complete the survey, potentially influencing the perceptions captured in the dataset. Evaluation primarily captured student self-reported perceptions rather than direct measures of anxiety, cognitive load, or in-class behaviour.

Qualitative feedback identified usability limitations that inform future development of the VR-Lab. Students reported navigation challenges, particularly when accessing the resource on smaller screens such as tablets or smartphones, suggesting that the VR-Lab is best experienced on a larger display. Feedback also indicated a desire for greater flexibility in navigating the resource, including the ability to revisit individual steps without restarting the activity. Future iterations should address these considerations to improve accessibility, usability, and learner control.

The comparison of summative assessment outcomes between cohorts was exploratory and based on anonymised historical data. Consequently, differences between cohorts cannot be attributed solely to the introduction of the VR-Lab, and assessment measures may be insensitive to changes in experiential aspects of learning. Future work should examine longitudinal implementation across multiple modules and cohorts, alongside evaluation approaches that explore in-class engagement and experiential outcomes more directly. Student feedback indicated that the VR-Lab reduced reliance on academic staff and enabled students to make more effective use of practical class time. This provides several potential measures for future evaluation including the number of staff interventions required, the number of procedural errors and the time taken to complete experimental set-up. Iterative refinement of the VR-Lab, particularly in relation to navigation and accessibility, will be important to maximise its effectiveness as a preparatory teaching resource.

## 5 Conclusion

This study suggests that virtual pre-laboratory resources can provide useful preparatory support for undergraduate pharmacology practicals by promoting student confidence, preparedness, and engagement with the laboratory environment. By reducing barriers associated with unfamiliar settings and procedures, the VR-Lab may contribute to supporting a more positive practical experience without replacing hands-on experimentation or directly influencing summative assessment outcomes. These findings support the use of low-cost, scalable virtual pre-laboratory activities as adjuncts to practical pharmacology teaching, particularly when designed to support inclusive access to experiential learning.

## Data Availability

The raw data supporting the conclusions of this article will be made available by the authors, without undue reservation.

## References

[B1] British Pharmacological Society (2021). Undergraduate Core Curriculum and Principles for Inclusive Delivery. London, United Kingdom: British Pharmacological Society.

[B2] Carriere J. J. Davies N. A. Cunningham M. R. Wallace M. J. Seeley A. (2023). Co-created *in vivo* pharmacology practical classes using the novel organism Lumbriculus variegatus. Pharmacol. Res. Perspect. 11 (6), e01158. 10.1002/prp2.1158 38063050 PMC10704400

[B3] Carroll J. S. Najafi H. Steiner M. (2025). Evaluating the effectiveness of virtual laboratory simulations for graduate-level training in genetic methodologies. Biochem. Mol. Biol. Educ. 53 (4), 422–432. 10.1002/bmb.21898 40417806 PMC12290252

[B4] Coyte E. (2023). Staff goals, challenges, and use of student inquiry in undergraduate bioscience teaching laboratories. FEBS Open Bio 13 (10), 1810–1830. 10.1002/2211-5463.13687 37548111 PMC10760353

[B5] Curry S. H. Whelpton R. (1983). Manual of Laboratory Pharmacokinetics: Experiments in Biopharmaceutics, Biochemical Pharmacology, and Pharmacokinetics, with a Consideration of Relevant Instrumental and Chromatographic Techniques.

[B6] Dempster J. (2020). ObSim: organ bath pharmacology simulation. Available online at: https://github.com/johndempster/ObSim/wiki (Accessed May 8, 2026).

[B7] Frei-Landau R. Avidov-Ungar O. Heaysman O. Ron-Ezra M. Schor Edelsztein H. Schwartz N. (2026). Blending video-based learning and simulations to practise inclusion of children with neurodevelopmental disorders. Technol. Pedagogy Educ., 1–26. 10.1080/1475939x.2026.2639404

[B8] George-Williams S. R. Blackburn R. A. Wilkinson S. M. Williams D. P. (2022). Prelaboratory technique-based simulations: exploring student perceptions of their impact on in-class ability, preparedness, and emotional state. J. Chem. Educ. 99 (3), 1383–1391. 10.1021/acs.jchemed.1c01116

[B9] Girmay S. Mendoza‐Franco G. Cardoso L. Jääskeläinen I. P. Kauppinen T. Nygren A. (2026). The impact of web-based 360° virtual laboratory on cognitive load, emotional patterns and visual attention. J. Comput. Assisted Learn. 42 (2), e70224. 10.1002/jcal.70224

[B10] Griffin C. S. Loughran S. Kelly B. Healy E. Lambe G. van Rossum A. (2025). Virtual laboratories complement but should not replace face-to-face classes: perceptions of life science students at Dundalk Institute of Technology, Ireland. Adv. Physiology Educ. 49 (2), 314–330. 10.1152/advan.00227.2024 39887303

[B11] Kirschner P. A. (1992). Epistemology, practical work and academic skills in science education. Sci. and Educ. 1 (3), 273–299. 10.1007/bf00430277

[B12] Kolb D. A. (2014). Experiential Learning: Experience as the Source of Learning and Development. Englewoods Cliffs, NJ: FT press.

[B13] Kurbanoglu N. Akin A. (2010). The relationships between university students' chemistry laboratory anxiety, attitudes, and self-efficacy beliefs. Aust. J. Teach. Educ. (Online) 35 (8), 48–59. 10.14221/ajte.2010v35n8.4

[B14] Rayamajhi S. Machin A. Breen C. Gebreheat G. Paterson R. (2024). Quality and impact of pharmacology digital simulation education on pre-registration healthcare students: a systematic literature review. Nurse Educ. Today 140, 106295. 10.1016/j.nedt.2024.106295 38959780

[B15] Salmon D. M. (2013). Practical Pharmacology for the Pharmaceutical Sciences. John Wiley and Sons.

[B16] Ural E. (2016). The effect of guided-inquiry laboratory experiments on science education students' chemistry laboratory attitudes, anxiety and achievement. J. Educ. Train. Stud. 4 (4), 217–227. 10.11114/jets.v4i4.1395

[B17] Wilhelmus M. M. Drukarch B. (2025). Hands-on practicals in pharmacology teaching at university level: outpaced by computer-based simulations, or is there a (blended) future? Eur. J. Pharmacol. 996, 177437. 10.1016/j.ejphar.2025.177437 40023358

