# Supplementary material for: Enhancing student confidence and preparedness in pharmacology: a virtual pre-laboratory approach
Source: Front Pharmacol. 2026 Oct 26;17:1897899. doi: 10.3389/fphar.2026.1897899 (PMC13745332; doi:10.3389/fphar.2026.1897899)
Supplement: Supplementary file 1 [file Supplementaryfile1.docx]

Supplementary Material

# 1 Post Practical Participation Survey Likert Questions (Strongly disagree - Strongly Agree)

1. I found the virtual lab engaging and intellectually stimulating
2. I found the virtual lab easy to navigate
3. The virtual lab reinforced what I learned in the introductory tutorial
4. I felt better prepared for the in-person lab sessions
5. I felt more confident during the laboratory practical's
6. The virtual lab is a good example of tech-enhanced learning
7. The virtual lab is a useful addition to the module

# 2 Post Practical Participation Survey Free Text Questions

1. What were the best aspects of the virtual lab?
2. What aspects of the virtual lab would you change?
3. Any other comments

**Supplemental Figures**

Supplemental Figure 1. Representative screenshots illustrating interactive elements within the VR‑Lab. (A) 360° laboratory environment with instructional overlay and interactive navigation. (B) Example of hotspot-based task progression requiring interaction with laboratory equipment in the correct sequence. (C) Integrated procedural guidance and inventory tracking during experimental activities. (D) Instructional overlay containing detailed protocol information and embedded demonstration video used to support student familiarisation with experimental techniques.

Supplemental Figure 2. Sequential workflow of VR-Lab. Students progressed sequentially through the virtual environment using interactive hotspots. Each stage incorporated procedural guidance, instructional content, embedded multimedia resources, and formative activities designed to familiarise students with equipment, experimental procedures, and expected outcomes.
